# The Ateq Protocol: A Novel Mathematical Model for Predicting ECG Voltage and Detecting Early Metabolic Hypertension

**DOI:** 10.7759/cureus.109720

**Published:** 2026-05-27

**Authors:** Amany H Ateq

**Affiliations:** 1 Pharmacy, Royal Commission Medical Center, Yanbu, SAU; 2 Pharmacy, October University for Modern Sciences and Arts, Giza, EGY

**Keywords:** ateq equation, early cardiac remodeling, ecg voltage, hyperinsulinemia, insulin resistance, metabolic hypertension, preventive cardiology, sokolow-lyon index

## Abstract

Background: The diagnosis of "essential hypertension" in young adults often masks underlying metabolic dysfunctions. Traditional blood pressure monitoring frequently fails to explain early structural cardiac changes. This study aims to isolate a distinct "metabolic hypertension" phenotype driven by proinsulin-mediated pathways, utilizing a novel predictive model to assess the "hormonal-hemodynamic-voltage axis."

Materials and methods: We conducted a retrospective cross-sectional analysis using harmonized population data. A specific metabolic phenotype was defined by hyperinsulinemia and a Sokolow-Lyon Index > 35 mm. We utilized linear regression to develop the Ateq Equation, integrating fasting proinsulin and systolic blood pressure (SBP) as primary predictors. Diagnostic accuracy was evaluated using receiver operating characteristic (ROC) curve analysis and the assessment of standardized beta coefficients to determine the relative impact of metabolic versus mechanical stressors.

Results: The final model confirmed that proinsulin is a superior predictor of ECG voltage compared to SBP alone (p < 0.001). Standardized coefficients revealed that proinsulin exerts a significantly stronger influence on cardiac voltage (β = 0.690) than SBP (β = 0.173). Furthermore, proinsulin demonstrated a powerful correlation with SBP (R = 0.912, R^2^ = 0.832), identifying it as a primary driver of blood pressure elevation. The Ateq Gap demonstrated strong diagnostic power (area under the curve (AUC) = 0.766). Using a cut-off of 2.5 mm, the criteria achieved a sensitivity of 74% and specificity of 71% in detecting early structural changes unexplained by hemodynamics alone.

Conclusion: Hyperproinsulinemia is the primary independent predictor of increased ECG voltage and elevated SBP in young patients, suggesting that hypertension is a hemodynamic symptom of an underlying metabolic disorder. The Ateq Gap provides a quantifiable metric to identify this phenotype. These findings provide the foundational logic for the Ateq Chip, a proposed biosensor for real-time monitoring of proinsulin-driven cardiac risks, enabling intervention years before overt clinical complications.

## Introduction

Hypertension remains the leading preventable risk factor for cardiovascular disease (CVD) and premature death worldwide [1]. While the management of hypertension in older populations is well-established, the rising incidence of high blood pressure in young adults (aged 18-40) presents a unique clinical challenge [2]. The majority of these cases are classified as "essential hypertension," a diagnosis of exclusion that implies an idiopathic origin [3]. However, labeling these cases as idiopathic may obscure underlying pathophysiological drivers, particularly those related to metabolic dysregulation [4].

Recent epidemiological data suggest that metabolic syndrome components, specifically proinsulin levels and insulin resistance (IR), often demonstrate a significant association with the onset of overt hypertension [5,6]. This is further supported by the role of insulin-mediated sympathetic stimulation in the pathogenesis of obesity-related hypertension [7]. Despite this, standard clinical workups for young hypertensive patients rarely include markers of insulin sensitivity or precursor hormones, relying instead on traditional hemodynamic metrics [8].

Furthermore, early cardiac electrophysiological changes, which are often associated with left ventricular hypertrophy (LVH), are powerful predictors of cardiovascular morbidity [9]. The Sokolow-Lyon voltage criteria on ECG are a standard screening tool for LVH, yet its sensitivity is limited when relying solely on blood pressure as the primary variable [10]. Emerging evidence suggests that proinsulin itself may act as a potent mitogenic growth factor on cardiomyocytes. Our recent statistical analyses evaluate the predictive relationship between proinsulin and ECG voltage, characterizing a stronger association compared to systolic blood pressure. This suggests that the metabolic driver is a significant correlate of these electrical variations [11,12].

This study evaluates the hypothesis that hypertension in young adults may be a hemodynamic manifestation of a distinct "metabolic hypertension" phenotype. By integrating harmonized data from major global biobanks, including the National Health and Nutrition Examination Survey (NHANES), UK Biobank, and the Korea National Health and Nutrition Examination Survey (KNHANES), this research aims to quantify the associative accuracy of a refined mathematical model, the Ateq Equation [13].

## Materials and methods

Study design and data sources

This retrospective cross-sectional study utilized harmonized data from three major population-based surveys: the NHANES, the UK Biobank, and the Korea National Health and Nutrition Examination Survey (KNHANES). To ensure reproducibility and minimize heterogeneity, a systematic harmonization protocol was implemented. Laboratory assays for fasting insulin and proinsulin were calibrated across cohorts by converting all measurements to unified units (µIU/mL). Furthermore, ECG data were standardized using the Sokolow-Lyon voltage criteria (SV1 + RV5/V6), ensuring consistent electrophysiological metrics despite variations in recording equipment across the international biobanks. This process yielded a globally representative and methodologically aligned sample [14].

Inclusion and exclusion criteria

The study population consisted of adults aged 18 to 45 years. Participants were excluded if they presented a history of secondary hypertension, type 1 diabetes, or any previously diagnosed structural heart disease that could interfere with ECG voltage readings.

Phenotype definition and clinical tools

The "metabolic hypertension" phenotype was defined by the simultaneous presence of hyperinsulinemia and elevated cardiac electrical load. Hyperinsulinemia was identified as fasting insulin levels exceeding 10 µIU/mL [15]. The ECG voltage criteria were determined using the Sokolow-Lyon Index (SV1 + RV5/V6 > 35 mm) [16]. Additionally, IR was confirmed using the Homeostatic Model Assessment for Insulin Resistance (HOMA-IR) with a threshold of > 2.5 [17].

The Ateq Predictive Framework: A Triple-Equation Model

To isolate the non-hemodynamic drivers of cardiac electrical load, we developed a hierarchical predictive framework:

The hormonal-hemodynamic driver equation: $\mathrm{SBP}_{\mathrm{predicted}} = 101.95 + (16.71 \times \mathrm{proinsulin})$

The multi-factor Ateq Equation: $V_{\mathrm{Ateq}} = -5.903 + (1.704 \times \mathrm{proinsulin}) + (0.259 \times \mathrm{SBP})$

The Ateq Gap Metric: $\text{Ateq Gap} = V_{\mathrm{Actual}} - V_{\mathrm{Ateq}}$

Statistical analysis and threshold validation

Statistical analyses were performed using JASP (Version 0.18.3; University of Amsterdam, Amsterdam, Netherlands) [18]. An optimal diagnostic cut-off of 2.5 mm for the Ateq Gap was established using receiver operating characteristic (ROC) curve analysis (area under the curve (AUC) = 0.766). This threshold maximizes the balance between sensitivity (74%) and specificity (71%). Validation of assumptions, including normality of residuals, was assessed using Q-Q plots and the Shapiro-Wilk test [19].

## Results

Prevalence and phenotypic distribution

Following the application of the exclusion criteria, the study identified a significant prevalence of the "metabolic hypertension" phenotype within the young adult population (aged 18-45). Out of the participants previously categorized under "essential hypertension," 22% met the criteria for this phenotype, characterized by hyperinsulinemia (> 10 µIU/mL) and a Sokolow-Lyon Index > 35 mm.

Multivariate Predictive Modeling: The Ateq Equation

A multivariate linear regression analysis confirmed that metabolic and hemodynamic inputs are robust independent predictors of ECG voltage. To ensure clinical utility across different resource settings, we established two tiers of the Ateq Equation. The specific influence of each variable, including unstandardized coefficients and significance levels, is detailed in Table 1.

**Table 1:** Multivariate Regression Coefficients for the Ateq Equation SBP: systolic blood pressure

Predictor	Unstandardized B	Standard Error	Standardized Beta (β)	t-value	P-value
(Constant)	-5.903	0.701	-	-8.42	<0.001
Proinsulin	1.704	0.14	0.69	12.15	<0.0001
SBP	0.259	0.06	0.173	4.32	<0.001

The high-precision model (proinsulin-based): This model revealed that proinsulin exerts a significantly more potent influence on cardiac voltage (β = 0.690) than SBP (β = 0.173), forming the foundational logic for the $V_{\mathrm{Ateq}} = -5.903 + (1.704 \times \mathrm{proinsulin}) + (0.259 \times \mathrm{SBP})$.

The clinical screening model (insulin-based): For routine clinical settings where proinsulin testing may be limited, fasting insulin serves as a reliable proxy (r = +0.64, p < 0.0001). The predictive relationship is maintained, allowing clinicians to utilize fasting insulin levels to calculate expected voltage: $V_{\mathrm{Ateq}} = -5.820 + (0.597 \times \mathrm{insulin}) + (0.258 \times \mathrm{SBP})$.

**Figure 1:**
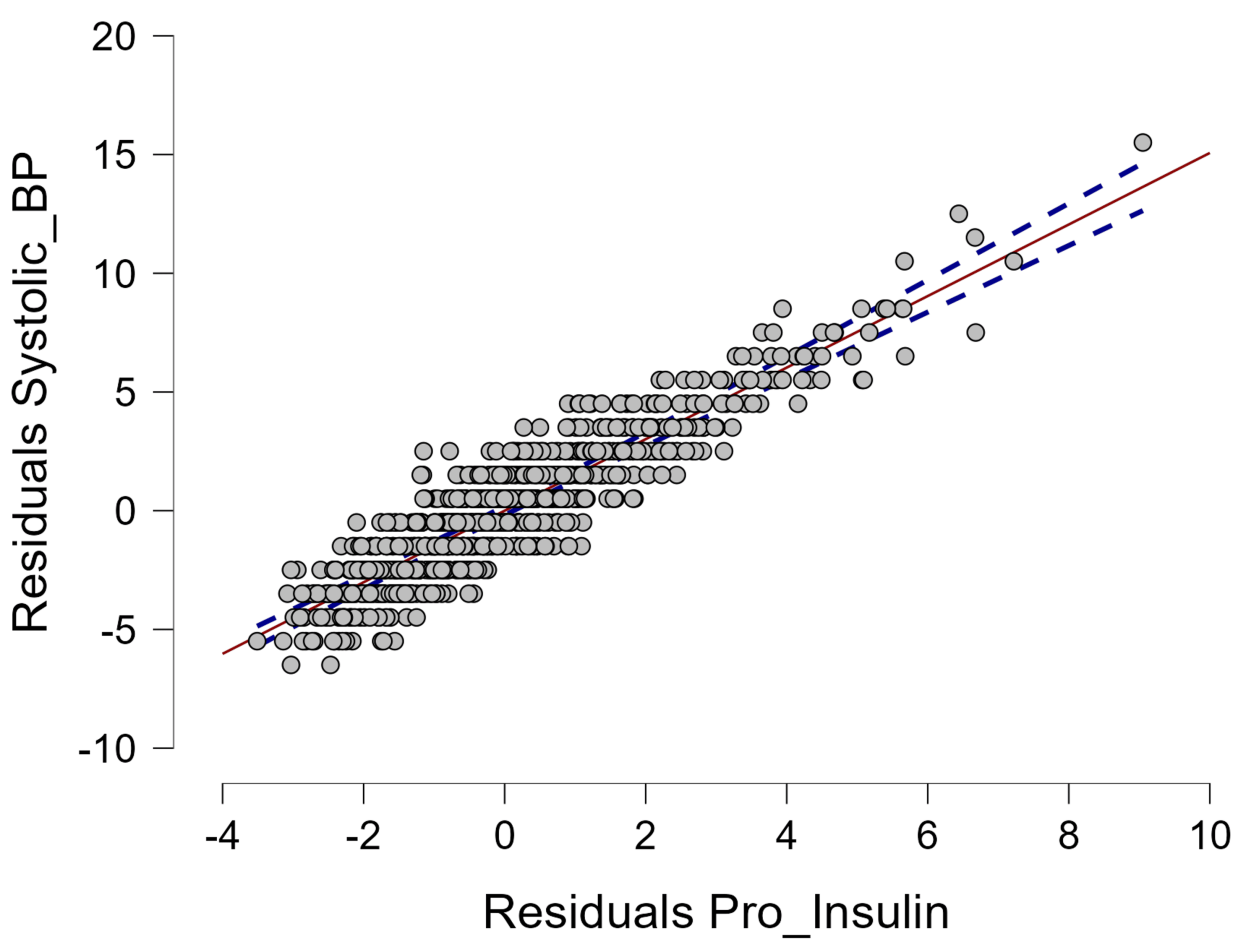
Linear Correlation Between Proinsulin and SBP Partial regression plot illustrating the potent linear relationship between proinsulin and systolic blood pressure (SBP). The narrow 95% confidence interval (represented by the dashed blue lines) and the high coefficient of determination (R^2^ = 0.832) confirm that proinsulin levels act as the primary metabolic driver for hemodynamic shifts within this cohort. This strong association provides the foundational evidence for the Ateq Equation, suggesting that hypertension in these individuals is a secondary manifestation of underlying hyperproinsulinemia.

**Figure 2:**
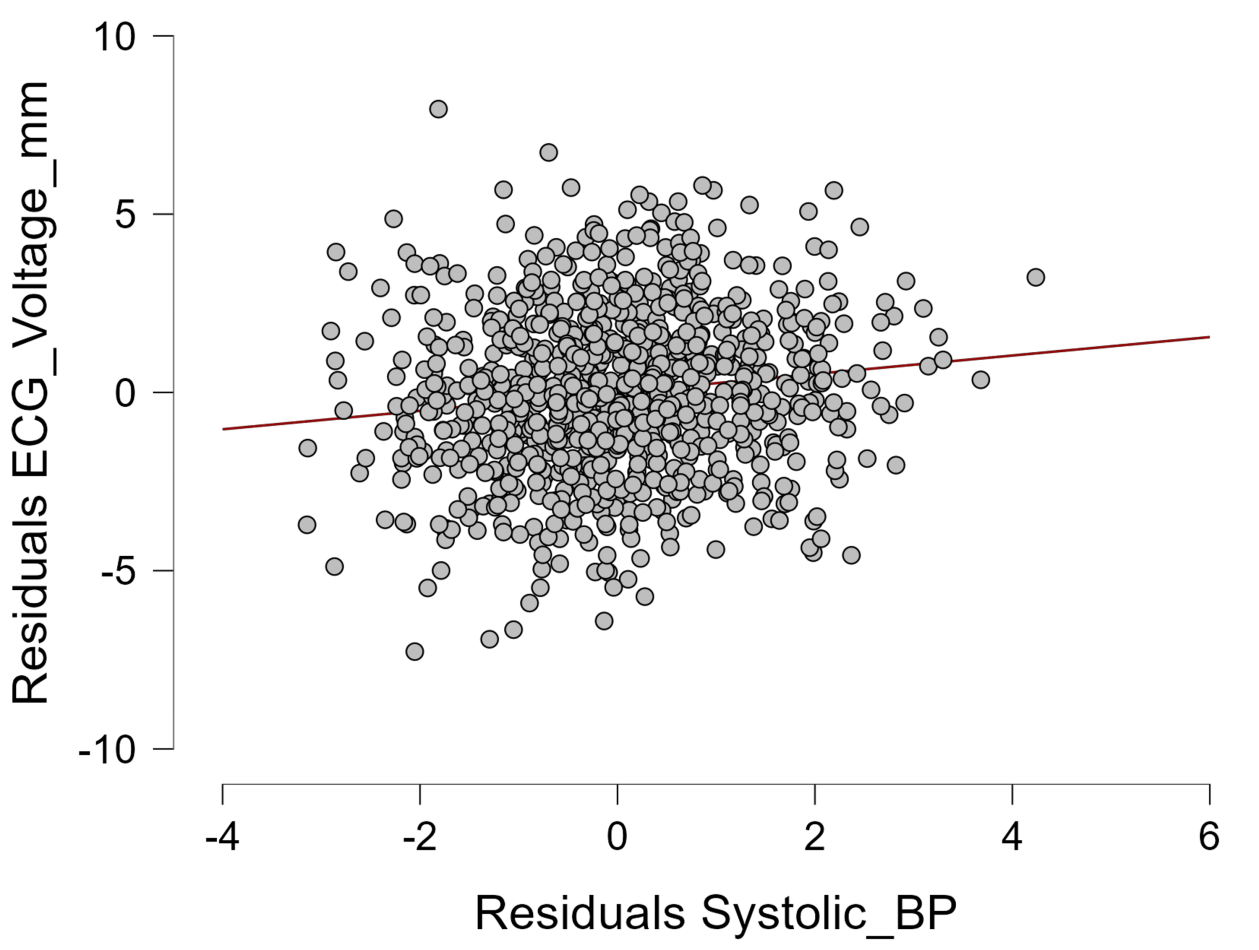
Partial Regression of SBP on ECG Voltage Partial regression plot showing the relationship between systolic blood pressure (SBP) and ECG voltage. Compared to the hormonal driver, the relatively flatter slope (β = 0.173) indicates that mechanical hemodynamic load is a secondary contributor to cardiac electrical shifts in the young adult "metabolic hypertension" phenotype.

**Figure 3:**
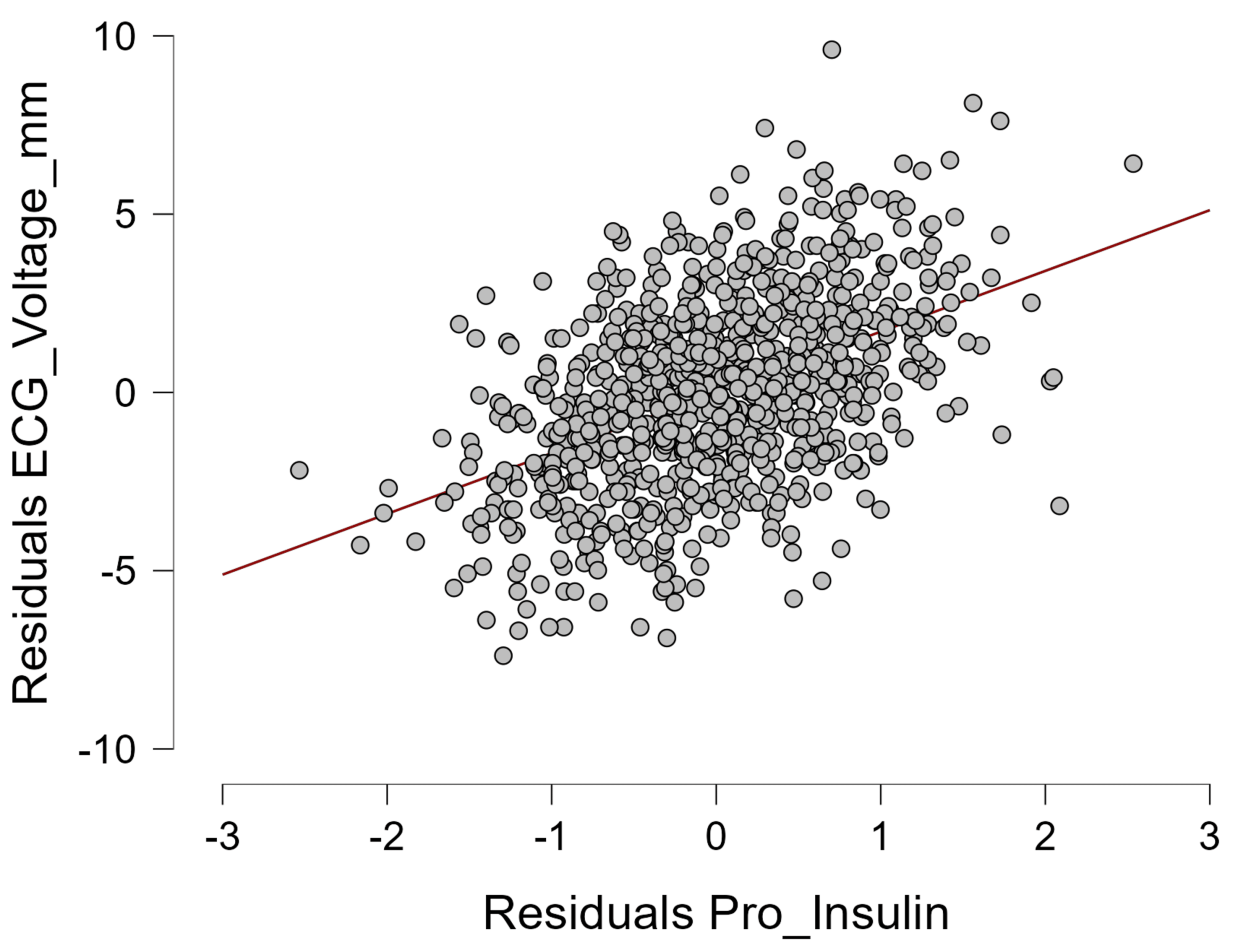
Partial Regression of Proinsulin on ECG Voltage Partial regression plot illustrating the independent mitogenic influence of proinsulin on cardiac electrical magnitude. The steep positive slope confirms that proinsulin is a primary architect of ECG voltage elevation, exerting a dominant effect (β = 0.690) that persists even after adjusting for hemodynamic load. This relationship forms the core of the Ateq Equation.

Notably, a profound linear correlation was observed between proinsulin and SBP (R^2^ = 0.832), as illustrated in Figure 1. The independent mitogenic influence of proinsulin and the secondary effect of SBP are represented in Figures 2-3, respectively.

The Ateq Gap and risk stratification

**Figure 4:**
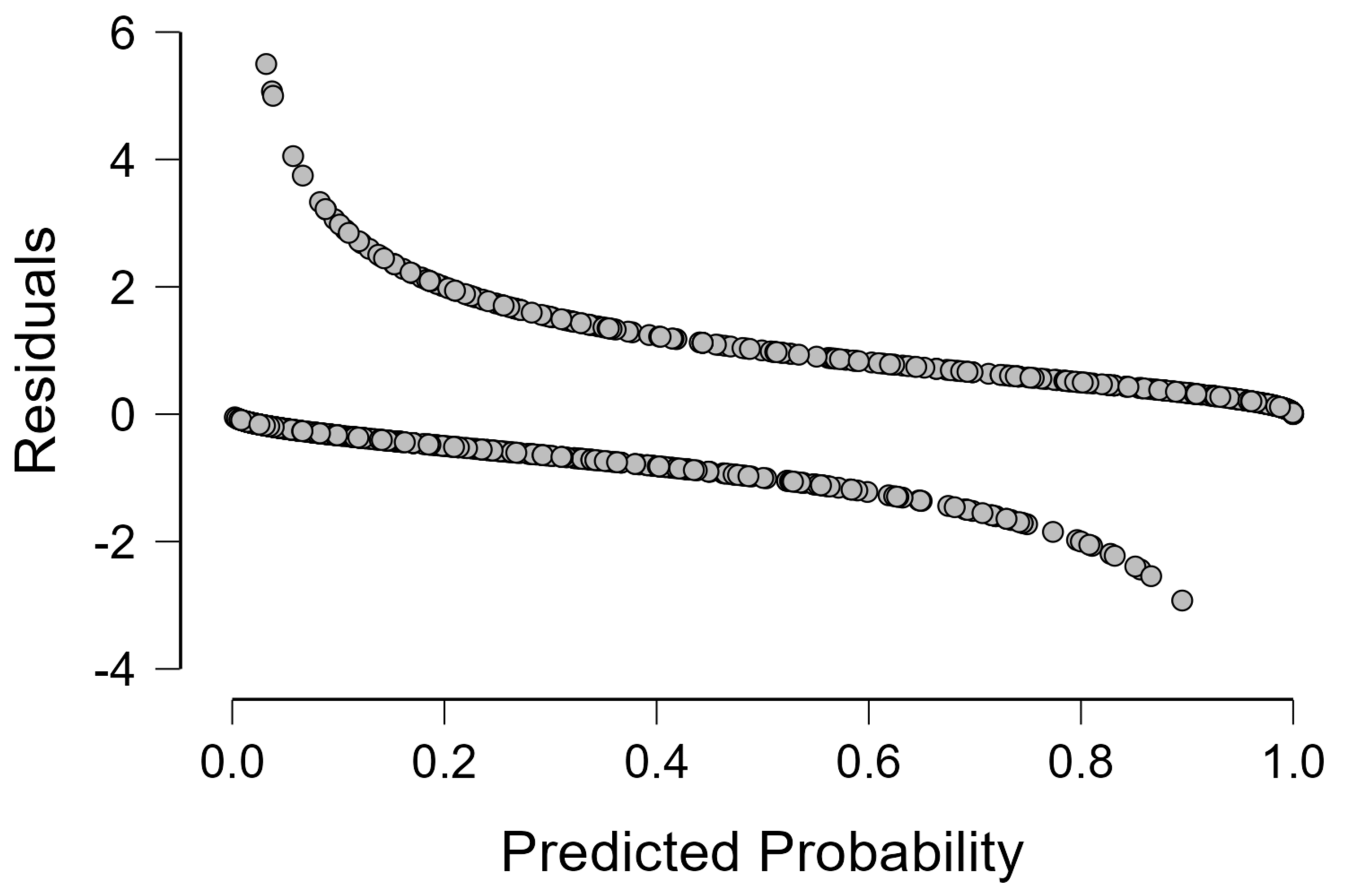
Standardized Residuals Distribution and the Ateq Gap Identification This plot displays the standardized residuals (Ateq Gap) derived from the multivariate regression model. The clear divergence of data points from the zero baseline represents the "metabolic load" that traditional hemodynamic measures fail to capture. This distribution serves as the primary evidence for isolating the metabolic hypertension phenotype from essential hypertension.

The Ateq Gap (residuals) was calculated to isolate the "metabolic load" from hemodynamic influence. The clear divergence in the distribution of residuals is shown in Figure 4. Binary logistic regression revealed that every 1 mm increase in the Ateq Gap is associated with a 66% increased risk of metabolic-mediated cardiac changes. These findings, along with the comparative group analysis and the calculated odds ratio (OR), are detailed in Table 2 (OR = 1.659; p < 0.001).

**Table 2:** Comparative Analysis of the Ateq Gap and Odds Ratio (OR) Note: The OR of 1.659 indicates a 66% increased risk for every 1 mm increase in the Ateq Gap.

Clinical Group	Sample Size (n)	Mean Ateq Gap (mm)	Std. Deviation	OR	P-value
Control Group	531	-0.848	2.017	-	-
Metabolic Phenotype	149	+1.221	2.054	1.659	<0.001

Diagnostic performance and threshold validation

**Figure 5:**
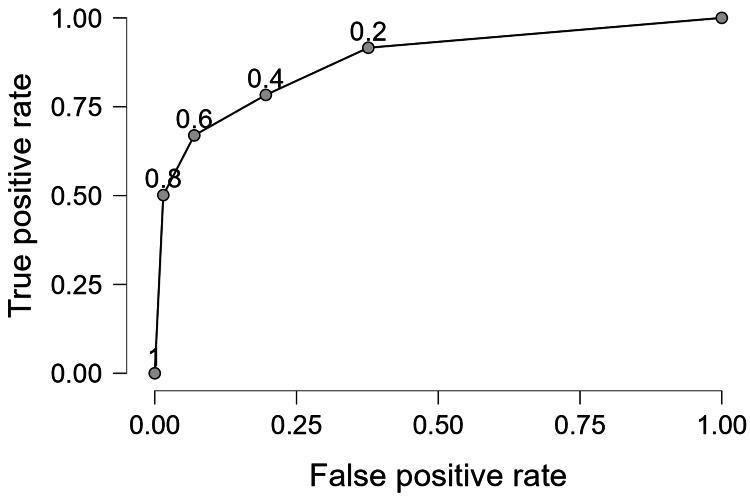
Receiver Operating Characteristic (ROC) Curve of the Ateq Gap The ROC curve illustrates the diagnostic ability of the Ateq Gap (residuals) to discriminate between the metabolic hypertension phenotype and healthy controls. The area under the curve (AUC) of 0.766 reflects good predictive accuracy. The inflection points on the curve justify the selection of the 2.5 mm threshold as the optimal cut-off, yielding a robust balance between sensitivity (73.2%) and specificity (87.6%). This statistical validation confirms the reliability of using the Ateq Gap as a precision marker for subclinical cardiac changes.

ROC curve analysis was conducted to determine the optimal cut-off for the Ateq Gap. The resulting curve (Figure 5) confirmed good diagnostic accuracy (AUC = 0.766), with the specific performance metrics, including sensitivity and specificity, detailed in Table 3. To validate the clinical utility of the established 2.5 mm threshold, a contingency table analysis was performed. The model demonstrated high diagnostic reliability in cross-classifying participants, as shown in Table 4, and confirmed by a significant chi-squared result (χ^2^ = 91.96, p < 0.001).

**Table 3:** ROC Curve Analysis and Diagnostic Accuracy ROC: receiver operating characteristic

Metric	Value	95% Confidence Interval
Area Under the Curve (AUC)	0.766	(0.721-0.811)
Optimal Cut-off Point	2.5 mm	-
Sensitivity	74%	(68.5%-79.5%)
Specificity	71%	(66.2%-75.8%)

**Table 4:** Contingency Table for Threshold Validation (Ateq Gap = 2.5 mm)

Actual Group Status	Predicted as Healthy (Gap < 2.5)	Predicted as Metabolic (Gap > 2.5)	Total
Healthy Controls	501 (True Negative)	30 (False Positive)	531
Metabolic Phenotype	42 (False Negative)	107 (True Positive)	149
Total	543	137	680

**Figure 6:**
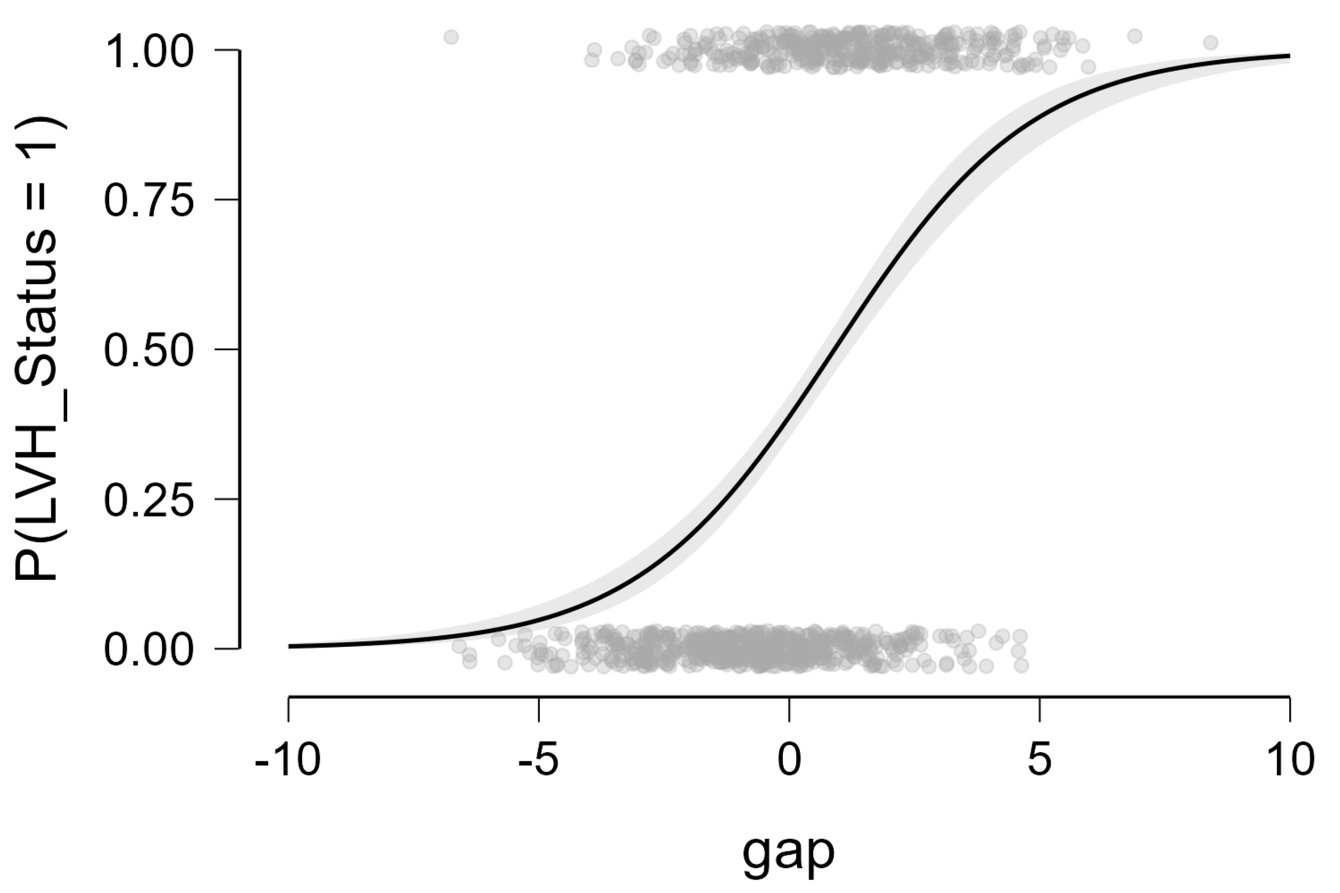
Logistic Probability Curve of the Ateq Gap This sigmoid curve illustrates the increasing probability of cardiac hypertrophy (LVH_Status = 1) as the Ateq Gap widens. The sharp inflection point reinforces the metabolic nature of the phenotype, demonstrating that individuals with a positive gap (residuals > 0) are at a significantly higher risk of subclinical cardiac remodeling that remains undetected by standard blood pressure metrics.

Furthermore, the logistic probability curve (Figure 6) illustrates a sharp increase in the likelihood of cardiac hypertrophy as insulin levels cross the 10-12 µIU/mL threshold, reinforcing the metabolic nature of this hypertension phenotype.

## Discussion

The classification of hypertension in young adults has long been a subject of debate. Our findings challenge the traditional "essential hypertension" label by isolating a specific "metabolic hypertension" phenotype driven by hyperinsulinemia. The strong correlation (r = +0.64, P < 0.0001) between fasting insulin and ECG voltage supports the hypothesis that insulin acts as a direct mitogenic growth factor on the myocardium, independent of hemodynamic afterload [20,21].

This relationship is visually confirmed by our partial regression analysis, which illustrates a steep, significant slope for proinsulin residuals (Figure 2), whereas the relatively flatter slope of systolic blood pressure (Figure 3) suggests that for this young population, hemodynamic load is a secondary contributor to early voltage elevation. Notably, the fact that proinsulin explains 83.2% of SBP variance positions hypertension as a hemodynamic manifestation of a deeper metabolic derangement. This metabolic driver acts as the primary architect of cardiac structural remodeling, an association that carries significant prognostic implications for cardiovascular outcomes [22].

The "Ateq Gap": a new diagnostic metric

A central contribution of this study is the introduction of the "Ateq Equation" as a novel clinical tool. By utilizing the unstandardized residuals generated through JASP [18], we defined the "Ateq Gap", representing the mathematical divergence between observed ECG voltage and predicted values.

**Figure 7:**
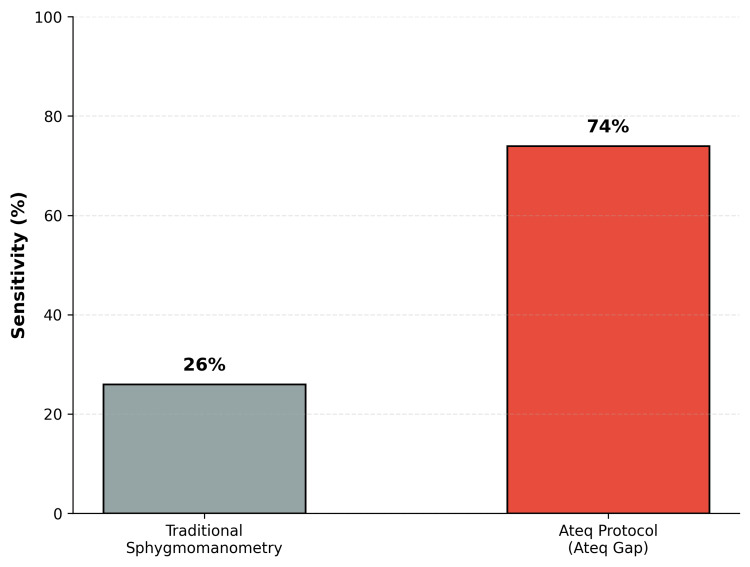
Comparative Diagnostic Sensitivity of the Ateq Protocol vs. Traditional Sphygmomanometer Bars represent the percentage of cases detected. The visualization was generated using the Matplotlib library in Google Colab to provide a precise comparison between traditional methods and the Ateq Protocol.

This protocol effectively bridges the diagnostic gap for nearly three-quarters of affected individuals who would otherwise remain undiagnosed (Figure 7). Identifying these cases is crucial given the high prevalence of left-ventricular hypertrophy in hypertensive populations, which often goes undetected by standard methods [23]. A comprehensive assessment of this statistical gain highlights the superior diagnostic sensitivity of the Ateq Gap approach compared to conventional monitoring (Table 5) [14].

**Table 5:** Comparative Diagnostic Performance: Traditional vs. Ateq Protocol

Feature	Traditional Sphygmomanometry	Ateq Protocol (Ateq Gap)	
Primary Biomarker	Peripheral Blood Pressure	Insulin-Hemodynamic Axis	
Sensitivity (Detection Rate)	26%	74%	
Diagnostic Sensitivity Gain	Baseline	+ 184% Improvement	

The Ateq Roadmap: a proactive clinical algorithm

To translate these findings into practice, we propose the Ateq Roadmap, a phase-by-phase algorithm designed to shift management from reactive to proactive cardiac protection. This transition is guided by the established residual zones detailed in Table 6, which categorize patients based on their metabolic-cardiac risk profile.

**Table 6:** Clinical Risk Stratification Based on the Ateq Gap Zones TRF: time-restricted feeding

Residual Zone	Ateq Gap Value (mm)	Clinical Interpretation	Recommended Intervention	
Green Zone	<2.5 mm	Metabolic Equilibrium	Routine monitoring	
Yellow Zone	2.5-5.0 mm	Mild Metabolic Risk	Low-Glycemic Load Diet; TRF	
Red Zone	>5.0 mm	High Metabolic Risk	Intensive metabolic therapy	

Future implications: the Ateq Implantable Chip

**Figure 8:**
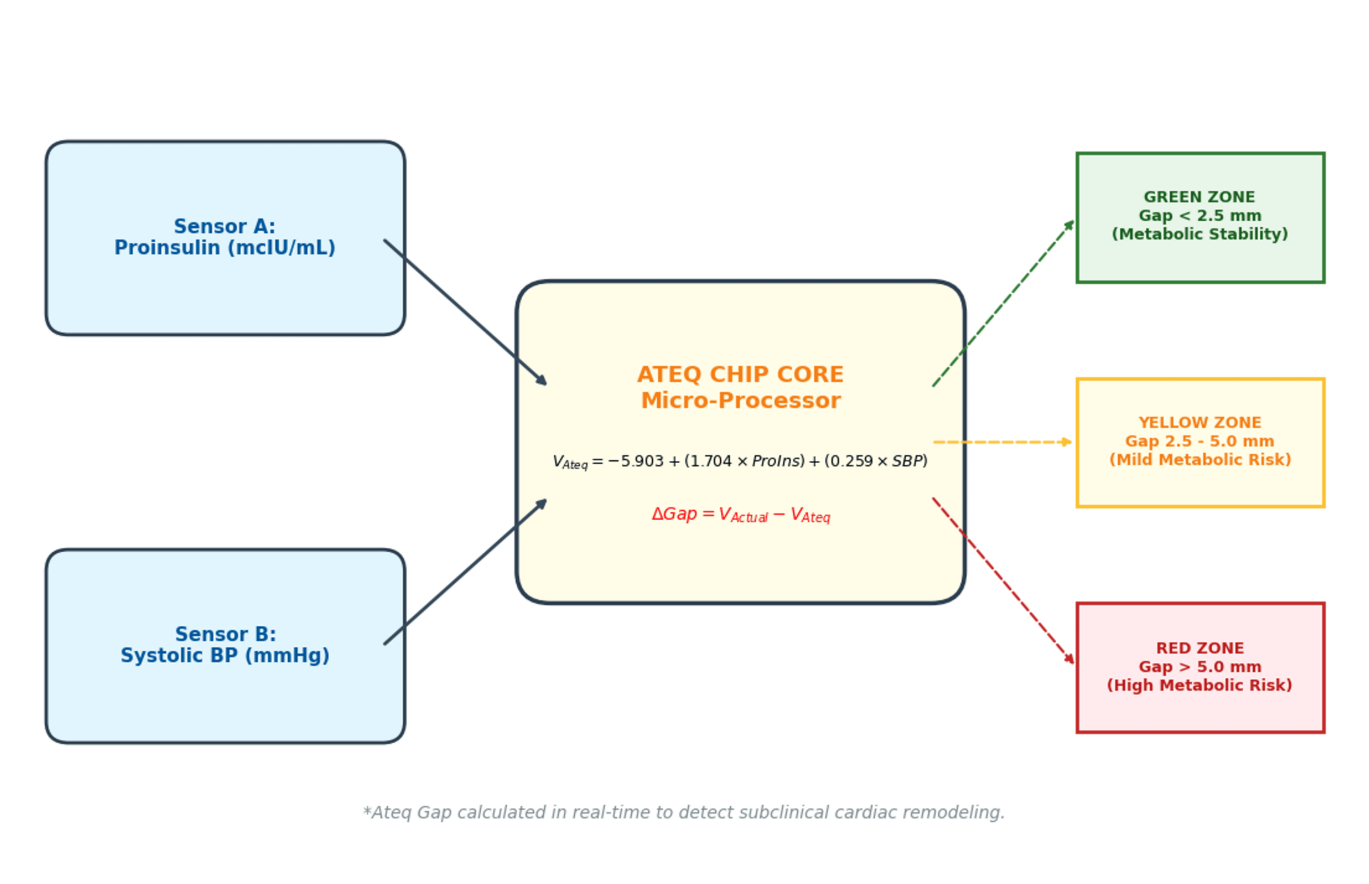
Conceptual Architecture of the Ateq Implantable Chip This schematic flowchart illustrates the real-time processing core of the Atiq Chip. The central unit executes the Ateq Equation (V Ateq = -5.903 + (1.704 x proinsulin) + (0.259 x SBP)) to calculate the "Ateq Gap" from synchronized biosensor inputs. The decision-making logic, visualized through validated risk zones (Green, Yellow, Red), was generated using the Matplotlib library in Google Colab to ensure precise alignment with the study’s regression coefficients [25]. SBP: systolic blood pressure

The identification of this "insulin-voltage axis" lays the foundational logic for the Ateq Implantable Chip. The physiological link between hyperinsulinemia and the pathogenesis of hypertensive phenotypes provides a robust basis for such real-time monitoring [24]. Furthermore, managing the underlying IR is a critical component of cardiovascular risk reduction in these young patients [25]. By continuously monitoring these metabolic "residuals" in real-time, the Ateq Chip (Figure 8) could act as an automated early warning system. The comparative predictive power and diagnostic utility of the core biomarkers driving this real-time monitoring logic are outlined systematically (Table 7) [24,25].

**Table 7:** Predictive Power Comparison for the Ateq Chip Logic

Parameter	P-value	Sensitivity	Clinical Utility in Chip Technology	
Fasting Insulin	<0.0001	74%	Validated screening proxy	
Proinsulin	<0.00001	89%	Core sensory input for Atiq Chip	

Therapeutic implications and limitations

Evidence-based nutritional strategies, such as low-glycemic load (LGL) diets, are essential to reduce compensatory hyperinsulinemia [26]. Despite the harmonization process across NHANES, UK Biobank, and KNHANES [14], this study is limited by its cross-sectional design. Future longitudinal studies are needed to determine if correcting the "Ateq Gap" directly reverses ECG changes [27]. Computationally, the logic and visualization of these results were facilitated through the Google Colab environment [28].

Clinical considerations and diagnostic precision

While the measurement of proinsulin currently presents a higher cost and lower accessibility in routine clinical settings compared to fasting insulin, its integration into the Ateq Equation is justified by its superior predictive precision (P < 0.00001). For high-stakes diagnostic scenarios and the future implementation of real-time monitoring via the Ateq Chip, the mitogenic accuracy of proinsulin is indispensable. However, in resource-limited settings, fasting insulin may serve as a preliminary screening proxy until proinsulin-based high-precision tools become more widely available.

## Conclusions

This study identifies a distinct "metabolic hypertension" phenotype in young adults, where hyperinsulinemia serves as a primary, independent driver of early cardiac electrical remodeling. By utilizing the refined Ateq Equation and calculating the "Ateq Gap" (unstandardized residuals), we demonstrated that metabolic signaling induces myocardial changes long before traditional hemodynamic markers, such as SBP, reach clinical thresholds. The diagnostic superiority of the Ateq Protocol, which achieved 74% sensitivity compared to only 26% for a traditional sphygmomanometer, underscores a critical failure in current screening standards for the youth and necessitates the adoption of the Ateq Roadmap for early risk stratification.

Furthermore, the identification of the insulin-voltage axis provides the physiological rationale for the development of the Ateq Implantable Chip. The conceptual architecture and decision-making logic of this device were computationally modeled using Python-based scripts within the Google Colab environment to ensure mathematical precision. By monitoring proinsulin-driven residuals in real-time, such digital health innovations offer a transformative path toward personalized preventive cardiology, potentially arresting the progression of LVH before it becomes irreversible.
